# Decoupling Polarization of the Golgi Apparatus and GM1 in the Plasma Membrane

**DOI:** 10.1371/journal.pone.0080446

**Published:** 2013-12-02

**Authors:** Blaine Bisel, Martino Calamai, Francesco Vanzi, Francesco Saverio Pavone

**Affiliations:** 1 European Laboratory for Non-linear Spectroscopy (LENS), University of Florence, Sesto Fiorentino, Italy; 2 National Institute of Optics, National Research Council of Italy (CNR), Florence, Italy; 3 Department of Evolutionary Biology “Leo Pardi”, University of Florence, Florence, Italy; University of Pecs Medical School, Hungary

## Abstract

Cell polarization is a process of coordinated cellular rearrangements that prepare the cell for migration. GM1 is synthesized in the Golgi apparatus and localized in membrane microdomains that appear at the leading edge of polarized cells, but the mechanism by which GM1 accumulates asymmetrically is unknown. The Golgi apparatus itself becomes oriented toward the leading edge during cell polarization, which is thought to contribute to plasma membrane asymmetry. Using quantitative image analysis techniques, we measure the extent of polarization of the Golgi apparatus and GM1 in the plasma membrane simultaneously in individual cells subject to a wound assay. We find that GM1 polarization starts just 10 min after stimulation with growth factors, while Golgi apparatus polarization takes 30 min. Drugs that block Golgi polarization or function have no effect on GM1 polarization, and, conversely, inhibiting GM1 polarization does not affect Golgi apparatus polarization. Evaluation of Golgi apparatus and GM1 polarization in single cells reveals no correlation between the two events. Our results indicate that Golgi apparatus and GM1 polarization are controlled by distinct intracellular cascades involving the Ras/Raf/MEK/ERK and the PI3K/Akt/mTOR pathways, respectively. Analysis of cell migration and invasion suggest that MEK/ERK activation is crucial for two dimensional migration, while PI3K activation drives three dimensional invasion, and no cumulative effect is observed from blocking both simultaneously. The independent biochemical control of GM1 polarity by PI3K and Golgi apparatus polarity by MEK/ERK may act synergistically to regulate and reinforce directional selection in cell migration.

## Introduction

Cell polarization and cell migration are interrelated, highly coordinated processes that allow complex, stratified tissue morphology and guided navigation in response to chemical cues [1]–[4]. In humans, cell polarization and motility are integral to essentially all higher order biological functions including the immune response [5]–[7], embryogenesis, neuronal development [8]–[12] and wound healing [13], [14], and play an important role in disease, most notably during cancer metastasis [15]–[17]. During cell migration, key structures including the actin network, mitochondria, the microtubule organizing center, the Golgi apparatus, and plasma membrane all polarize to support locomotion [1], [3], [4], [18]. GTPases including Ras, Raf and Cdc42 synchronize these polarization events through complex and highly regulated signaling cascades [19]–[23].

The Golgi apparatus, a central sorting hub involved in protein and lipid synthesis, modification, and secretion [24]–[26], was among the first organelles suspected to play a role in cell polarization and migration [27], [28] The Golgi apparatus becomes oriented, along with the centrosome, in front of the nucleus and facing the leading edge or principal membrane protrusion in most types of polarized or migrating cells including epithelial cells, fibroblasts, lymphocytes, and neurons. Because of the central role of the Golgi apparatus in membrane homeostasis and secretion, it is thought to supply either general or specialized membrane components to the leading edge of polarized cells [29]–[32]. Blocking Golgi apparatus polarization toward the leading edge inhibits cell motility [33]–[35]. Disrupting Golgi cargo vesicles through various strategies, including brefeldin A (BFA) or monensin drug treatment, protein kinase D knock down, or microinjecting the ARF1-Q71L constitutively active mutant, prevent the development of morphological features of polarization such as lamellipodia or dendrite outgrowth [34]–[37].

Another critical event in cell polarization is the development of asymmetry in the plasma membrane. Membrane microdomains, sometimes called lipid rafts, have been implicated in early stages of cell polarization and shown to be important for migration as well [38]–[40]. Membrane microdomains are detergent-resistant subregions of the plasma membrane enriched in cholesterol, sphingolipids, transmembrane signaling proteins, receptors, and associated adaptor proteins [41], [42]. These microdomains, which have been reported to range in size from 25 to 700 nm, contribute to the accumulation of growth factor receptors and associated signaling molecules, increasing signaling efficiency [41]. Membrane microdomains, when accumulated in a polarized fashion, also contribute to the creation of intracellular signaling gradients that are central to cell polarization [38], [43]. GM1 is an important component of membrane microdomains in many cell types which is synthesized in the Golgi apparatus [44].

The Golgi apparatus, and more specifically, the trans-Golgi network (TGN) is thought to play an important role in sorting of glycolipids and associated GPI-anchored proteins and contributing to their asymmetric accumulation in polarized cells [42], [45]–[47]. However, several Golgi-independent mechanisms have been proposed to contribute to polarization of lipid raft components in the plasma membrane. These mechanisms include self-assembly, or clustering, of membrane rafts via receptor crosslinking [48], actin-mediated crosslinking and stabilization [49], microtubule-based active transport [43], recycling pathways including clathrin [50] or clathrin-independent caveolar recycling [51], and a BFA-insensitive exocytic pathway that bypasses entirely the Golgi apparatus [52].

Previous studies of cell polarization have taken advantage of tracking experiments in which the localization of a molecule of interest is monitored to assess the polarity of its distribution [35], [43], [48]. To bridge the gap between the molecular and the cellular organelle levels, we were interested in assessing the polarization of the Golgi apparatus and GM1 on a cell by cell basis. The geometric complexity and variability of both the Golgi apparatus and plasma membrane has often led to qualitative analysis methods. Here we devise improved image analysis methods that allow for objective and quantitative analysis of labeled structures within the cell. With these methods, we are able to measure the polarization of the Golgi apparatus and GM1 simultaneously in individual cells while assessing the effects of different drugs that disrupt pathways essential to cell polarization. Applying statistical analysis methods to polarization measurements in individual cells allowed us to detect differences unable to be observed in standard ensemble analysis.

Here we demonstrate that GM1 in the plasma membrane polarizes before the Golgi apparatus just 10 min after stimulation, while both structures result to be polarized after 30 min. By using drugs that block Golgi polarization or function, GM1 polarization is shown to develop independently from Golgi apparatus polarization. Golgi apparatus polarization is additionally shown to progress even when GM1 polarization is inhibited. The decoupling of Golgi apparatus and plasma membrane polarization appears to be the result of separate intracellular pathways controlled by Ras/Raf/MEK/ERK and PI3K/Akt/mTOR, respectively. We confirm the independent nature of Golgi and GM1 polarization through correlative analysis of coupled measurements in single cells. Lastly, we examine the roles of Golgi apparatus and GM1 polarization in the context of cell migration and invasion in healthy and cancerous cell types. Inhibiting Golgi apparatus polarization produces a greater inhibition of two-dimensional migration, while inhibiting GM1 polarization decreases the efficiency of three dimensional cell invasion, and, in both cases, there is no cumulative effect from blocking both.

## Results

### Quantifying Golgi apparatus and GM1 polarization

An accurate quantification of polarization is essential to investigate the relationship between the Golgi apparatus and GM1 in the plasma membrane, and allows us to determine the individual contributions of each structure, define the effects of various drugs, and assess correlation between the two events.

**Figure 1:**
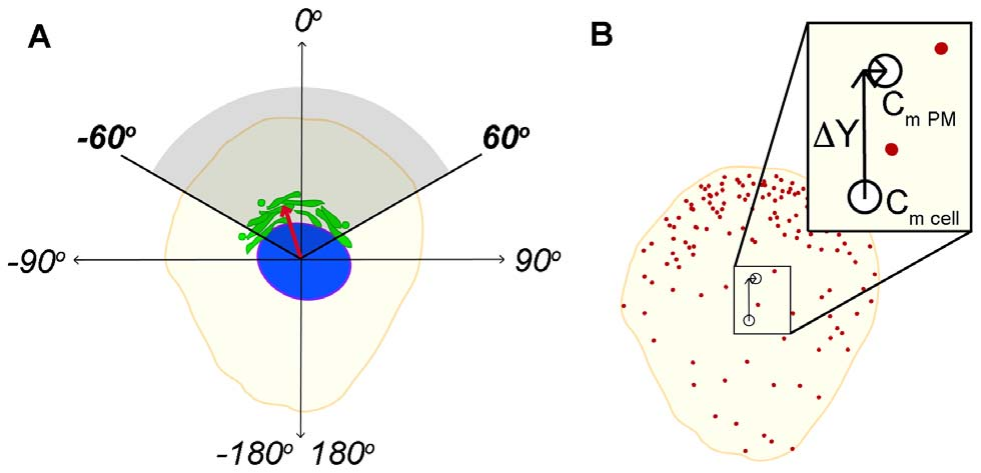
Quantification of Golgi apparatus and GM1 polarization. (**A**) Golgi apparatus polarization was calculated as a vector (shown in red) that connects the center of mass of the nucleus (C_m nuc_.) to the center of mass of the Golgi (C_m Golgi_). From this Golgi vector, an angle is calculated with reference to the *y* axis, defined as 0° and facing the wound edge. When compared to the classical method, angles falling from −60° to +60° are the equivalent of an oriented Golgi falling within a 120° angle facing the wound edge (shaded area). (**B**) Plasma membrane polarization was determined by comparing the weighted fluorescence distribution of QDs in the plasma membrane with the geometric center of mass of the cell. GM1 gangliosides in the plasma membrane were labeled with cholera toxin subunit B conjugated to QDs, represented here as red circles. The weighted center of mass of GM1 QD fluorescence was calculated (C_m PM_) along with the geometric center of mass of the cell (C_m cell_). Subtracting the *y* component of C_m cell_ from the *y* component of C_m PM_ gives us ΔY, a measure of the shift in plasma membrane polarization towards the scratch.

To induce cell polarization, we utilized a scratch assay [27], [34], [53], [54], which allows precisely-timed measurements of the early stages of cell polarization upon stimulation with growth factors [54]. Golgi apparatus polarization is commonly measured based on a quartile or tertile scheme in which a circular grid is placed over the nucleus of a cell of interest and oriented toward the defined direction of polarization, i.e. perpendicular to the scratch [22], [27], [53], [55]–[57]. If the Golgi apparatus, or a majority of the Golgi apparatus, falls within the quartile or tertile facing the direction of polarization, it is defined as oriented, or polarized. This type of Golgi orientation test allows an accurate quantification of the percent of cells with polarized Golgi within a population, but conveys a binary value of *oriented* or *not oriented* for each individual Golgi, a limiting factor that results from the inherently binned measurement. We were interested in developing a quantitative, continuous measurement of Golgi polarization that would allow us to analyze relative increases or decreases in polarization. As in the original method, Golgi polarization was defined relative to the axis running from the nucleus to the scratch. We calculated a vector that connects the center of mass coordinates of the nucleus to the center of mass coordinates of the Golgi (Figure 1A). The positive *y* direction was defined as 0°, which is perpendicular to the scratch and corresponds to an oriented position of the Golgi apparatus. Using this system, Golgi angles in the range from −60°to +60° are equivalent to the classical 120° angle facing the wound edge.

Assessing plasma membrane polarity has been previously achieved with the use of fluorescently labeled membrane components like GFP-tagged or antibody-labeled membrane receptors [58]–[60] or lipid based optical probes that can, for example, differentiate between liquid ordered or disordered phases of membrane lipids [61], [62]. Analysis of these labeling methods has usually relied on quantifying ratios of fluorescence labeling at different areas of a polarized plasma membrane, such as from front to back in a polarized cell or across a membrane subjected to a polarization-inducing gradient [38]. Recently, an assay for plasma membrane polarization based on single molecule labeling of γ-aminobutyric acid (GABA) receptors with QDs in neural growth cones has been described [43]. We have built upon this methodology and modified the assay to fit whole cell measurements in our wound model system. GM1 in the plasma membrane was labeled by biotinylated cholera toxin subunit B conjugated to streptavidin Quantum Dots (QDs). GM1 is synthesized in the Golgi apparatus [44] and is transported to the plasma membrane during cell polarization [63]. Although the dynamic behavior of GM1 does not represent that of all polarizing molecules of the plasma membrane, GM1 gangliosides are widely regarded as constituents of membrane rafts [7], [41], [64], and have been shown to distribute toward the leading edge during cell polarization [65], making GM1 a good candidate for studying membrane polarity. We measured the intensity-weighted center of mass of the GM1-QD fluorescence distribution in the plasma membrane for a single cell (C_m PM_). We then calculated the center of mass of the cell using the coordinates of all pixels defining the cell profile (C_m cell_). Subtracting the y component of the C_m cell_ from the y component of the C_m PM_ determined the shift in center of mass towards the leading edge due to QD fluorescence, with the result designated as ΔY (Figure 1B). ΔY is expressed in µm, where the positive *y* direction is defined as facing the wound. High values of ΔY, therefore, indicate an asymmetric accumulation of fluorescence towards the wound edge.

### GM1 polarizes before the Golgi apparatus

**Figure 2:**
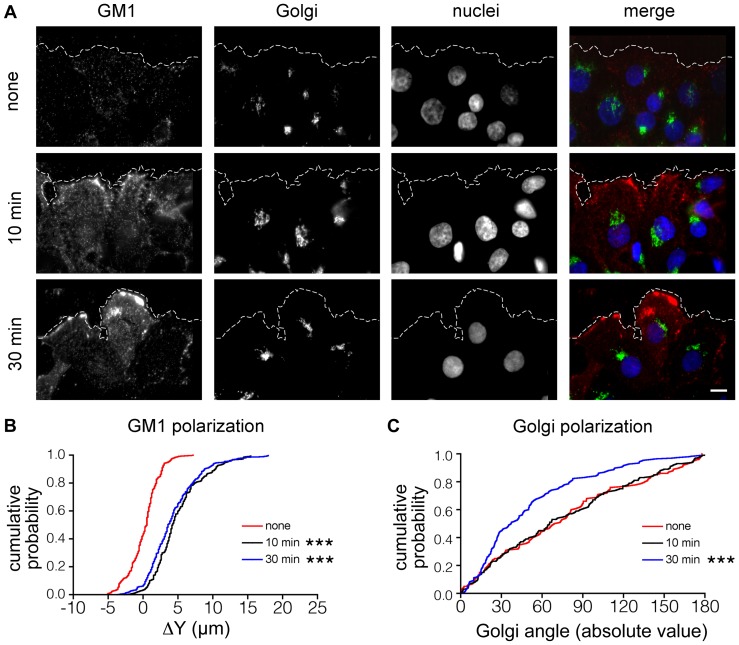
Golgi apparatus and GM1 polarization in response to LPA. (**A**) Composite images (red, GM1; green, Golgi; blue, nuclei) from untreated wound-edge cells (none) and wound-edge cells incubated with LPA for 10 min or 30 min (scale bar, 10 µm). (**B**) Cumulative distributions of ΔY plasma membrane polarization values. At both 10 min and 30 min stimulation, ΔY values are significantly increased with respect to control. None, n = 121; 10 min, n = 152, p = 0.001; 30 min, n = 225, p = 0.001. (**C**) Cumulative distributions of the absolute values of Golgi angles. Compared to control, the LPA-stimulated Golgi apparatus is polarized after 30 min but not after 10 min. None, n = 111; 10 min, n = 150, p = 0.945; 30 min, n = 116, p = 0.001. *** represents p≤0.001 compared to control using the Kolmogorov-Smirnov test.

Polarization of the Golgi apparatus and GM1 were initially tested upon stimulation with lysophosphatidic acid (LPA) for 30 min (Figure 2). Both showed significantly increased polarization compared to unstimulated controls. Cells at the wound edge were labeled with Cholera toxin-QD for GM1, anti-GM130 for the Golgi apparatus, and Hoechst staining for the nucleus. The lower panels B and C display the measured values of GM1 and Golgi apparatus polarization, calculated in individual cells, as cumulative distribution functions. Kolmogorov-Smirnov non-parametric tests were used to assess significance between conditions because the results of both Golgi apparatus and GM1 polarization were determined to be non-normally distributed. The effect of the stimulation is clear at the population level, nevertheless it is notable that even in the LPA-stimulated population we observe a portion of cells that lack a polarized plasma membrane or Golgi apparatus. Still, we confirm that our model cell line shows a measurable and reliable polarization response to stimulation with LPA. Furthermore, a comparison of the Golgi polarization results obtained using the present semi-automated method and the standard by-eye method shows good agreement (Figure S1).

We also tested whether reducing the LPA stimulation time might reveal which structure polarizes first, giving further information about the underlying mechanisms and coordination between the two structures (Figure 2). We found that 10 min after stimulation with LPA, the level of GM1 polarization was comparable to that following 30 min stimulation, while Golgi apparatus polarization did not show a significant increase compared to unstimulated controls. These results indicate that since GM1 polarizes before the Golgi apparatus, it is unlikely that Golgi polarization is driving GM1 polarization in the plasma membrane.

### Golgi apparatus and GM1 polarization depend on separate intracellular pathways

**Figure 3:**
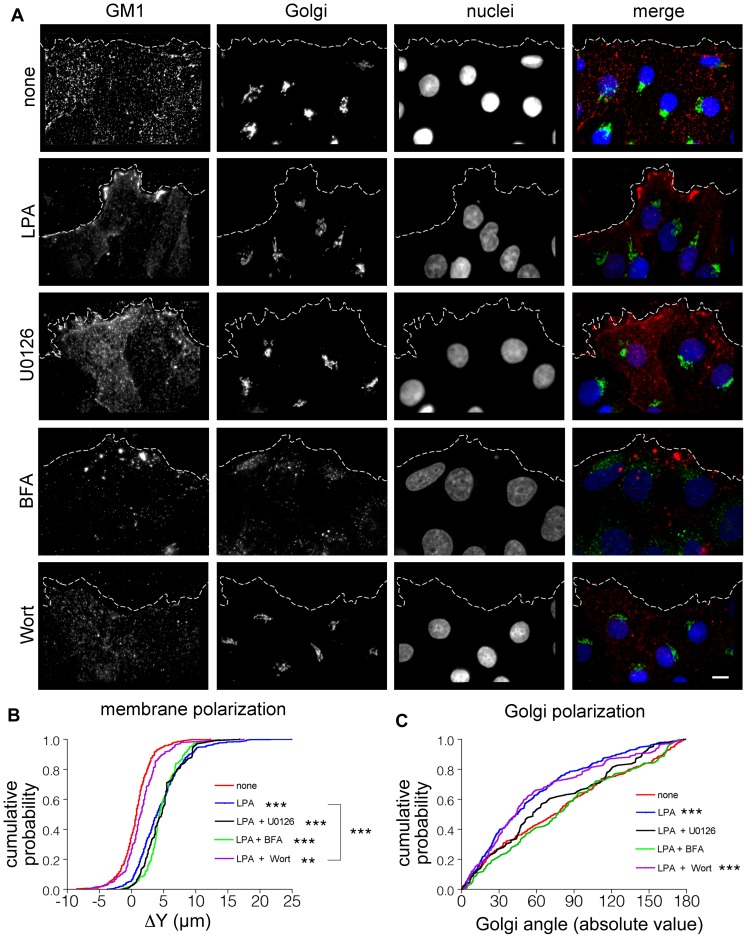
Drug treatments reveal uncoupled pathways to Golgi apparatus and GM1 polarization. (**A**) Composite images (red, GM1; green, Golgi; blue, nuclei) from control experiments (none), incubation with LPA for 30 min, and 30 min pretreatment with the drugs U0126, BFA, or Wortmannin before incubation with LPA (scale bar, 10 µm). (**B**) Comparison of cumulative distributions of ΔY plasma membrane polarization for all treatments. The plasma membrane is polarized in all cases compared to the unstimulated control, (none, n = 326; LPA, n = 323, p = 0.001; U0126, n = 87, p = 0.001; BFA, n = 104, p = 0.001; Wortmannin, n = 144, p = 0.003), but wortmannin incubation significantly inhibits polarization when compared to LPA incubation alone (LPA vs. Wort, p = 0.001). (**C**) Cumulative distributions of Golgi angles show that Golgi polarization is inhibited by BFA and U0126, but unaffected by wortmannin. None, n = 293; LPA, n = 322, p = 0.001; U0126, n = 87, p = 0.183; BFA, n = 104, p = 0.736; Wortmannin, n = 144, p = 0.001. *** represents p≤0.001 compared to control using the Kolmogorov-Smirnov test.

To confirm the assertion that Golgi polarization is not required for GM1 polarization, we decided to utilize a drug known to block Golgi apparatus polarization. U0126 interferes with the ERK-induced phosphorylation of the Golgi structural protein GRASP65, inhibiting the Golgi cisternal unstacking required for polarization [53]. A 30 min pretreatment with U0126 followed by 30 min stimulation with LPA in the continued presence of the drug resulted in an inhibition of Golgi polarization but had no effect on GM1 polarization (Figure 3). These results further support that the establishment of GM1 polarization does not rely on the polarization of the Golgi apparatus.

U0126 treatment does not inhibit membrane trafficking associated with the Golgi apparatus, so to address the possibility that vesicles emerging from the Golgi apparatus may still contribute to membrane polarization, we performed an experiment in the presence of brefeldin a (BFA). BFA reversibly inhibits Arf1 activity by binding to the Arf1-GDP-Sec7 complex, promoting conformational changes in Arf1 and ultimately preventing the exchange of GDP to GTP [66]. Arf1 and Sec7 are essential components of the COPI coat complex of vesicles budding from the Golgi apparatus and directed by retrograde transport to the ER [67], [68]. The cumulative effect of BFA treatment is dissociation of the Golgi apparatus, with Golgi enzymes redistributed into the ER and Golgi matrix proteins relocated to ER exit sites, and an inhibition of secretory traffic [69]–[72]. We found that BFA treatment left GM1 polarization intact (Figure 3B), indicating that Golgi trafficking does not contribute to the observed polarization of the membrane. Additionally, although the Golgi structure was largely disrupted by the drug, we were still able to measure residual Golgi polarization based on the distributions of the scattered punctate structures and found that, perhaps not surprisingly, what remained of the Golgi structure was not polarized.

Because we found no effect on GM1 polarization when interfering with various functions of the Golgi apparatus and because GM1 polarization in the plasma membrane was found to occur earlier in the development of cell polarization, we decided to ask whether blocking GM1 polarization had an effect on Golgi apparatus polarization. One idea is that spatial signaling is developed initially in the plasma membrane based on forces transmitted through cell adhesion interactions [73]. This leads to the formation of membrane protrusions and asymmetry at the leading edge, which are stabilized and maintained by Golgi polarization controlled by Cdc42. By inhibiting the pathway leading to GM1 polarization, we would expect to see a downstream effect on Golgi polarization if the two are linked either biochemically or mechanically. We utilized the drug wortmannin, which inhibits PI3K and thus the formation of phosphatidylinositol (3,4,5)-trisphosphate (PIP3) [74], which accumulates in the leading edge membrane of polarized cells [75]. We first asked whether PI3K inhibition would decrease the observed polarization of GM1 in the plasma membrane, and whether decreased GM1 polarization would affect Golgi apparatus polarization. We found that treatment with wortmannin blocked the polarization of GM1 but had no influence on Golgi apparatus polarization (Figure 3C). It appears, therefore, that the two polarization events of these key cellular compartments are not linked. The results are also not specific to stimulation with LPA. We performed additional experiments with epidermal growth factor (EGF) and fetal bovine serum (FBS) to stimulate polarization, yielding similar results (Figure S2). Taken together, this body of evidence suggests that two separate intracellular signaling pathways, the PI3K/Akt/mTOR pathway (blocked by wortmannin) and the Ras/Raf/MEK/ERK pathway (blocked by U0126), separately and independently control the polarization of GM1 and the Golgi apparatus.

### Simultaneous measurements of Golgi apparatus and GM1 polarization in single cells

**Figure 4:**
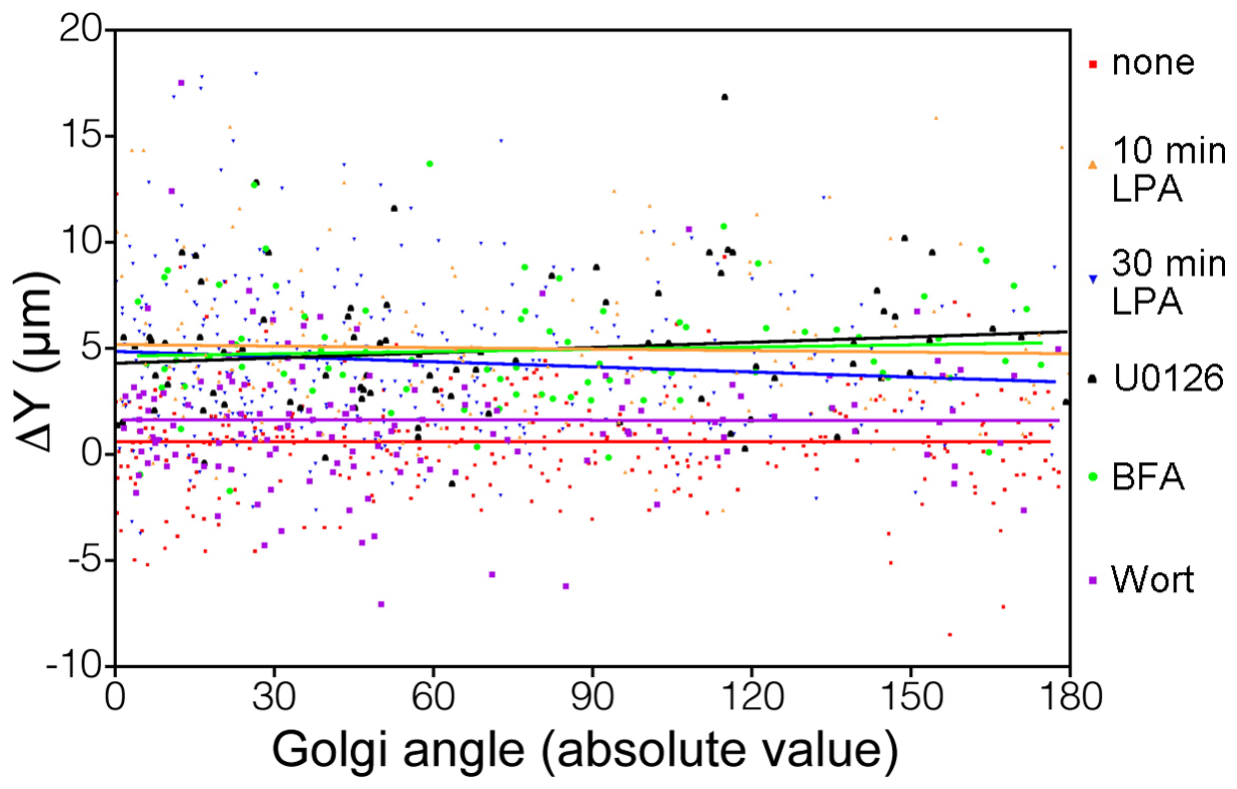
Correlation between ΔY and the Golgi angle in individual cells. The value of ΔY in µm and the absolute value of the Golgi angle are plotted on the *y* and *x* axes, respectively. One point corresponds to the value of ΔY and the Golgi angle calculated in a single cell. The Spearman's rank correlation coefficient ρ and associated p value are reported for each condition. None of the conditions has a significant correlation between GM1 polarization and Golgi apparatus polarization, and all slopes are near zero. None, n = 290, *ρ* = 0.01, p = 0.92; LPA 10 min, n = 150, *ρ* = −0.06, p = 0.44; LPA 30 min, n = 293, *ρ* = −0.08, p = 0.16; U0126, n = 87, *ρ* = 0.11, p = 0.32; BFA, n = 85, *ρ* = 0.06, p = 0.61; Wortmannin, n = 144, *ρ* = 0.01, p = 0.95.

Next, we tested whether any correlation could be determined between Golgi and GM1 polarization at the level of the individual cell. Using the two polarization measurements previously collected from single cells after no stimulation, stimulation with LPA for 10 min or 30 min, or pretreatment with U0126, BFA or wortmannin, we constructed scatter plots of paired Golgi apparatus and GM1 polarization measurements recorded from single cells (Figure 4). Spearman correlation analysis showed no significant correlation between Golgi apparatus and GM1 polarization. In other words, for any single cell, the state of polarization of either the Golgi apparatus or GM1 is not predictive of the other. These results further support the assertion that Golgi apparatus and GM1 polarization develop independently with separate biochemical and mechanical mechanisms.

### Analysis of GM1 distribution among experimental populations

**Figure 5:**
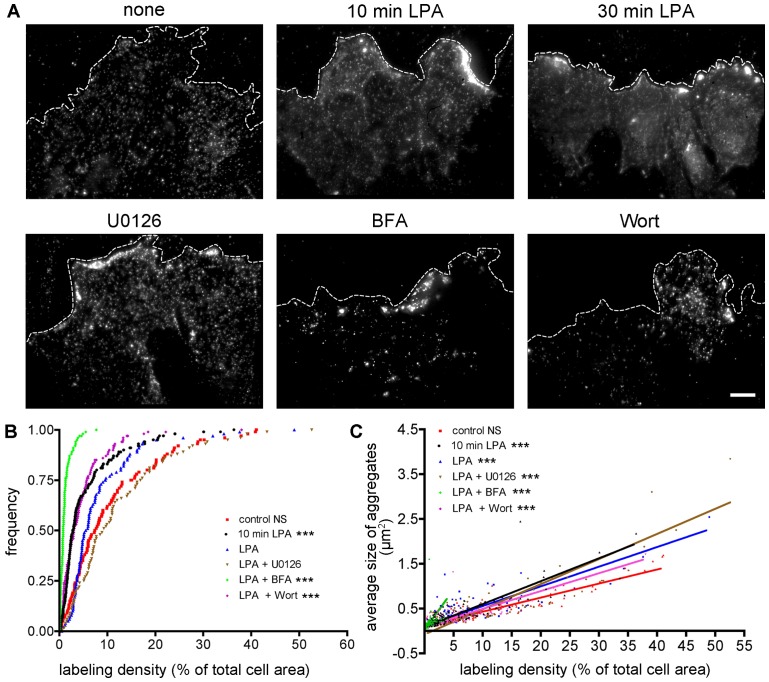
Analysis of GM1 distribution in response to drug treatments. (**A**) GM1 gangliosides labeled with cholera toxin-QDot conjugates at a wound edge after exposure to LPA stimulation in the presence of drugs blocking either GM1 polarization or Golgi apparatus polarization (scale bar, 10 µm). (**B**) Total amount of GM1 labeling for each condition shown as frequency distributions. The GM1 labeling density is the percentage of total cell area covered by fluorescence signal based on thresholded particle analysis. Each point represents the value for a single cell. *** represents p≤0.001 compared to control using Kolmogorov-Smirnoff statistical tests. (**C**) Clustering behavior analysis. The average size of aggregates per cell in µm^2^ was plotted against GM1 labeling density, and Deming (model II) linear regression was performed to fit the data and determine slope. Slopes and the associated 95% confidence interval were used to determine significance between experimental groups. *** represents p≤0.001 compared to control using Student's t tests. For (**B**) and (**C**), none, n = 111; LPA 10 min, n = 151; LPA 30 min, n = 107; U0126, n = 86; BFA, n = 120; Wortmannin, n = 144.

While assessing membrane polarization, we observed that the pattern of GM1 staining was highly variable not only between different experimental conditions, but also between cells of the same population (Figure 5). Levels of GM1 expression have been observed to vary up to 100 fold between cells of the same population [76]. Although the origin of this heterogeneity is not well understood, it has been shown to depend, in part, on the cell context within a population (e.g. dependent upon surrounding cell density and the relative position within cell islets) [77]. To explore how stimulation with LPA or pretreatment with the various drugs affected GM1 expression and the extent of labeling, we calculated the amount of GM1 labeling as a percentage of the total cell surface area (Figure 5B). The bottom quartile of the population sorted for expression levels ranged from 0.4% labeling density for BFA treated cells to 5.1% for U0126 treated cells. The top quartile ranged from 1.3% labeling density to 16.9% for BFA and U0126 treatments, respectively, representing the minimum and maximum labeling conditions. Within the same treatment condition, labeling density ranged from 0.1% to 7.7% for BFA and 0.5% to 52.6% for U0126, accounting for an approximate 100-fold difference in expression level within a given population. Kolmogorov-Smirnoff statistical tests revealed that, compared to the unstimulated control population, BFA, wortmannin, and 10 min stimulation with LPA had significantly reduced labeling density profiles.

The other pattern of GM1 labeling that we observed to vary among the treatment groups was the appearance of large clusters or aggregates. While clusters were occasionally observed in all experimental conditions, they seemed to appear more frequently in some conditions. Clusters concentrated at the leading edge were apparent in LPA-stimulated populations, contributing to the cell polarization measured in Figs. 2 and 3. On the contrary, unstimulated control cells appeared to have a more dispersed pattern of GM1 labeling. The difficulty of assessing GM1 clustering behavior was compounded by the fact that, as described above, the total amount of GM1 labeling displayed large variations between individual cells, even in the same experimental group (Figure 5B). With this variability in mind, we plotted the average size of aggregates as a function of labeling density to analyze the clustering behavior of GM1 (Figure 5C). As expected, increasing labeling density corresponds to increasing aggregate size. This is probably due to a combination of two factors. First, increased expression of GM1 may lead to increased membrane raft formation and clustering behavior. Because we have labeled cells with cholera toxin and QDots after fixation, the increased clustering behavior represents only the unlabeled, physiological behavior of GM1 and excludes the known effects of cholera toxin crosslinking in live cells. Second, apparent clustering would be expected to increase due to optical saturation as a result of increased GM1 labeling density. Although we are not able to separate these two possible causes of observed clustering, we can discern the differences between the treatment groups at any given labeling density. For example, although BFA treatment greatly reduces the total amount of labeling, for any given labeling density, BFA-treated cells have a much greater probability of aggregation, shown by the increased slope (green diamonds, Figure 5C). Cells treated with LPA also have a significantly greater propensity to show GM1 aggregation at any given concentration in comparison with unstimulated control cells. In the case of wortmannin-treated cells, the clustering behavior remains intact even though polarization has been inhibited, as demonstrated in Figure 3.

### Activation of both MEK/ERK and PI3K is required for efficient cell migration

**Figure 6:**
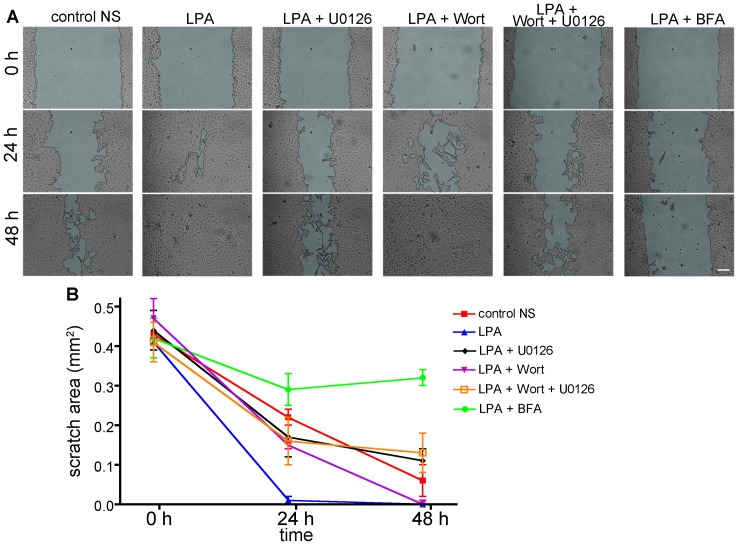
Cell migration wound closure assay after treatment with drugs. (**A**) ECV304 cells grown to confluence and starved overnight were scratched with a pipette tip, and treated with either the continued presence of starvation medium (none), LPA stimulation, or LPA stimulation and inhibitory drugs U0126, wortmannin, wortmannin and U0126 combined, or BFA. Images of the scratch were collected after 0, 24 and 48 hours. (**B**) The wound area was measured at 3–5 points along the scratch for each time point and results were pooled for three experiments. Two-way ANOVA and Bonferroni post-tests comparing all columns were used to assess significance. P values and N, representing the number of frames collected and analyzed, are reported in Tables S1–S3 in File S1. Scale bar, 100 µm.

We have described the acute effects of blocking Golgi apparatus and GM1 polarization via inhibition of the MEK/ERK and PI3K/AKT pathways, but we also wanted to explore the longer term consequences of inhibiting these pathways on cell migration. Using the inhibitors U0126, wortmannin, and BFA, we performed assays to measure both two and three-dimensional migration. First, in a wound closure assay, a confluent monolayer of cells scratched with a pipette tip and treated with the drugs either individually, or with a combination of U0126 and wortmannin were photographed after 0 h, 24 h, and 48 h (Figure 6). Quantification of the wound closure was achieved by calculating the area of the cell-free region over time. The wound areas measured before adding drugs (time point 0 h) were measured to range from 0.41±0.05 mm^2^ to 0.47±0.15 mm^2^, and most were not significantly different (Table S1 in File S1). We found that LPA-stimulated control cells nearly completely closed the wound after 24 h with an area remaining of 0.01±0.01 mm^2^, and had completely closed the wound after 48 h. Unstimulated control cells, which were nonetheless found capable of migration, were measured to have a mean 0.22±0.02 mm^2^ open area remaining after 24 h and 0.06±0.04 mm^2^ area remaining after 48 h. After 24 h, cells treated with either wortmannin, U0126 or a combination of wortmannin and U0126 were statistically indistinguishable (Table S2 in File S1). After 48 h, however, cells administered the combination of wortmannin and U0126 were observed to follow the same kinetics as U0126 alone, while wortmannin treated cells continued to completely close the wound with an area remaining of 0.00±0.01 mm^2^, indistinguishable from the LPA stimulated control (Table S3 in File S1). These results suggest that MEK/ERK activation (inhibited by U0126) may be the dominant factor in two-dimensional cell migration. BFA treatment shows minimal wound closure after 24 h and 48 h (0.29±0.04 mm^2^ and 0.32±0.02 mm^2^ of open wound areas, respectively). That BFA-treated cells show minimal wound closure is most likely due to the toxic effects of chronic inhibition of intracellular traffic, and also due to inhibition of cellular proliferation, which is a contributing factor towards wound closure in these timescales.

**Figure 7:**
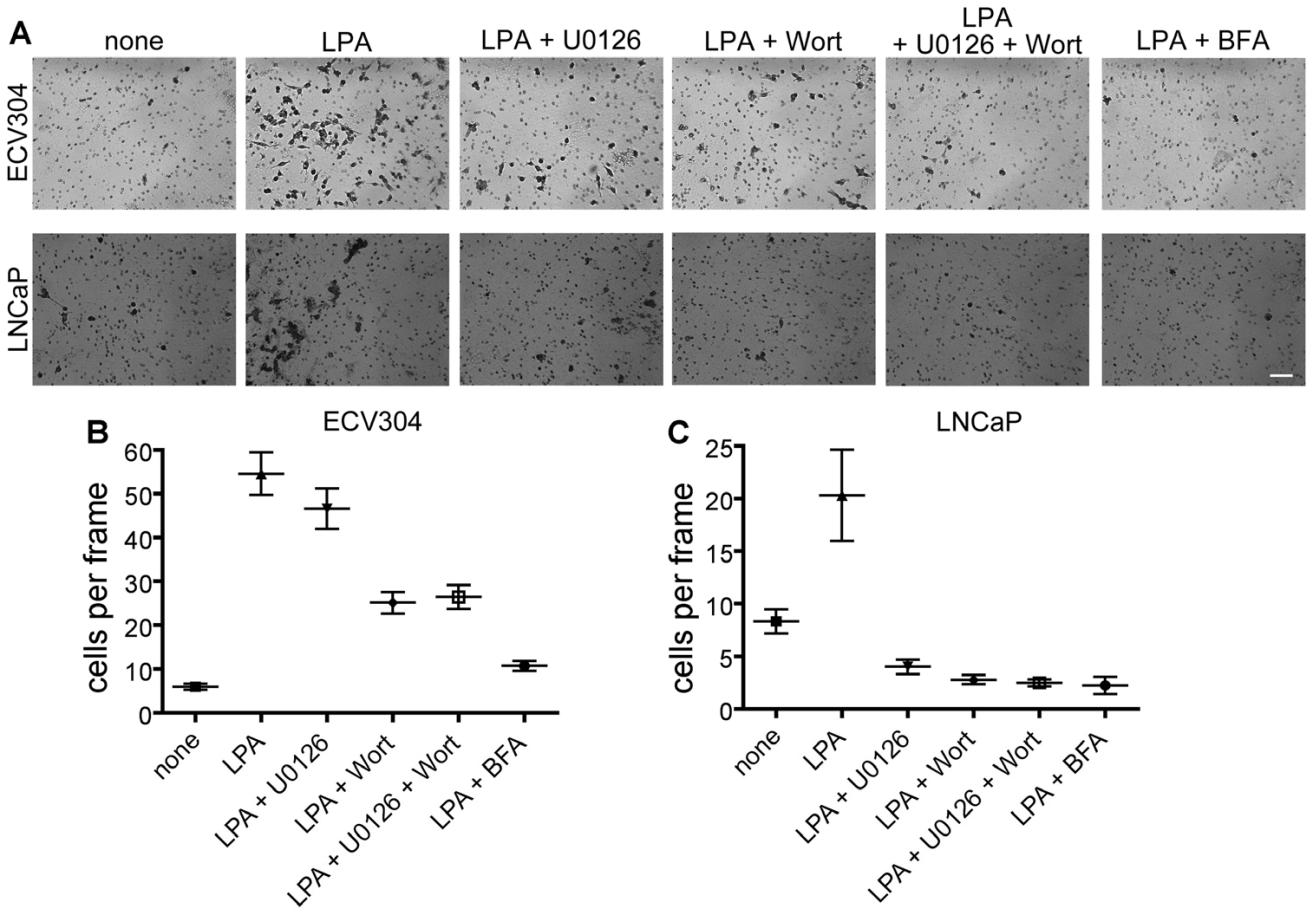
Matrigel invasion assay in the presence of drugs. ECV304 or LNCaP cells were counted and loaded into the upper chamber of a 0.8 µm pore insert containing matrigel basement membrane matrix. The lower chamber contained LPA chemoattractant, and both chambers contained inhibitors where indicated. Cells were allowed to migrate for 20 h before fixation and removal of unmigrated cells. Remaining migrated cells were stained to increase contrast, photographed and counted (**A**). The mean number of cells per frame was calculated from combined results from a minimum of 3 experiments (**B**) and (**C**). One-way ANOVA with Tukey post-tests to compare all columns was used to assess significance. The resulting p values and N, representing the number of frames used for analysis, are reported in Tables S4 and S5 in File S1. Scale bar, 100 µm.

Because the results of the two-dimensional wound closure assays at timescales of 24 to 48 h are a product of both cell migration and cell proliferation, we decided to probe the effects of the various drugs that block Golgi apparatus and GM1 polarization on three-dimensional cell migration during shorter timescales (Figure 7). A gelled matrix invasion assay allowed us to determine migration efficiency in a setting that mimics physiological conditions over a period of 20 h. Cells were allowed to migrate through a layer of matrigel basement membrane matrix gel and through 0.8 µm pores towards the chemoattractant LPA in a lower chamber. After 20 h, unmigrated cells were removed, and migrated cells were processed for microscopy. We evaluated cell migration by counting the number of cells per frame in images of the lower face of the porous membrane. Three-dimensional migration was assessed in ECV304 cells as well as in an invasive prostate cancer cell line, LNCaP, which were not suitable for testing by the wound closure assay as they do not form sheets. We found that, as expected, stimulation in the bottom chamber with LPA significantly increased the number of cells migrating through the matrix compared to unstimulated controls from a mean of 5.9±5.2 cells per frame to 54.6±35.1 cells per frame for ECV304 (Figure 7B) and from 8.3±6.6 cells per frame to 20.3±21.3 cells per frame for the LNCaP prostate cancer cell line (Figure 7C). In the LNCaP cell line, treatment with U0126, wortmannin, BFA, or combined U0126 and wortmannin resulted in a significant decrease in the number of migrated cells compared to LPA control, but differences between the drug treatments were indistinguishable (Table S5 in File S1). In ECV304 cells, U0126 treatment did not result in a significant decrease compared to LPA, but wortmannin, BFA, and a combined treatment of U0126 and wortmannin did significantly decrease the number of migrated cells compared to LPA alone (Table S4 in File S1). Interestingly, contrary to the pattern observed for two-dimensional migration, ECV304 cells showed statistically indistinguishable results for wortmannin treatment and combined wortmannin and U0126 treatment, suggesting that PI3K/AKT inhibition has a greater impact on three-dimensional cell migration. Consistent with the two-dimensional scratch assay, the combined effect of the drugs was not greater than with any individual drug, suggesting that inhibition of the two pathways does not produce a cumulative effect. BFA again shows the strongest inhibitory effect on cell migration, but this result could again be due to toxic effects of prolonged BFA exposure.

## Discussion

### Correlation of polarization events in single cells

We have described new methodologies for quantifying Golgi apparatus and GM1 distributions in polarizing cells. Previously, Golgi apparatus orientation has been quantified using an angular coordinate system superimposed upon cells at the scratch leading edge [22], [27], [53], [55], [57]. In these assays, a researcher determines by eye whether the majority of the Golgi apparatus falls within a predetermined arc of usually 120° or sometimes 90°, and the Golgi is marked as oriented or not oriented. While this system is simple to implement and generally gives reliable results, the major improvement of our new methodology is that measurements are automated, saving time, increasing accuracy, and reducing bias. Additionally, the output is no longer binned into one of two categories, but is continuous, allowing us to assign a continuous value of Golgi polarization to individual cells. A related assay yielding similar results was implemented by Yadav and colleagues to measure Golgi polarization on a radian scale which was based on measuring fluorescence intensity for each radian degree in a circle centered at the cell nucleus [31]. A notable improvement, however, in our analysis method is the implementation of cumulative distributions and the non-parametric Kolmogorov-Smirnoff test to highlight significant differences between experimental populations.

In addition, we measured the degree of polarization of the membrane raft component GM1 in the same cells where Golgi apparatus polarity was assessed. Polarization of the plasma membrane has been assessed in previous works by either qualitative visual categorization [33], [60] or comparing the ratio of front to rear fluorescence [61], [75]. The present method has been adapted from a study measuring the distribution of GABA receptors in cultured neurons stimulated with a GABA gradient using a center of mass based quantification [43]. While conceptually similar to methods presented in the aforementioned study, we made several technical modifications to record the QDot signal over the entire cell, including acquiring a series of z stacks.

By combining these two continuous measurements of polarity in the same cells, we were able to compare the degree of polarization of the two structures and thus assess trends and correlations both at the population level and within single cells. At the population level, we found differences in the timing of the development of polarization (Figure 2), with GM1 polarization developing after just 10 min and Golgi polarization after 30 min. This result is in agreement with reports that plasma membrane polarization develops as soon as 3 min [38] or 10 min [33], [43]. These membrane polarization times were measured, however, in neuronal growth cones exposed to growth factor gradients [38], [43] or epithelial cells exposed to an electric field [33]. Moreover, they studied the distribution of transmembrane receptors while we used the ganglioside GM1 as a polarization marker. Previous experiments that compared the polarization of the Golgi apparatus and membrane raft markers examined the specific case of the temperature sensitive VSVG viral glycoprotein that is synthesized in the ER and retained in the Golgi apparatus until the permissive temperature allows its transport to the plasma membrane within 30 min [29], [37], [52]. It is probably the case that distinct mechanisms account for the different polarization dynamics observed between membrane receptors, GM1, and VSVG, and these are discussed in more detail below.

In addition to the different timescales of polarization, we utilized the center of mass assays to test the effects of various drug treatments, revealing the presence of two separate intracellular pathways controlling Golgi and GM1 polarization. In the case of U0126, which inhibits MEK, the mechanism for inhibiting Golgi apparatus polarization was described previously to rely on phosphorylation of GRASP65 at serine 277 [53]. Phosphorylated GRASP65 occurs normally during mitosis and also cell migration, leading to Golgi cisternal unstacking [78]. This unstacking, which occurs prominently in mitosis and to a lesser degree in cell polarization, facilitates vesiculation, therefore allowing even distribution of Golgi membranes into daughter cells during mitosis, and also relocalization of the organelle during cell polarization. Blocking this phosphorylation with U0126 is thought to promote a hyperstabilization of the Golgi apparatus that lacks the plasticity needed to polarize. In this work, we used U0126 as a tool to block Golgi apparatus polarization without affecting its function, and found no inhibitory effect on the polarization of GM1 in the plasma membrane, suggesting that the two events are not linked.

We also treated cells with BFA, which induces rapid disassembly of the Golgi apparatus and a redistribution of Golgi enzymes into the ER and Golgi structural proteins including GM130 to ER exit sites (ERES) by reversibly inhibiting Arf1, a component of the COPI coat assembly [66]–[68]. Even in this dispersed state, we were able to measure the cumulative polarization of the GM130-labeled Golgi remnants, since our method relies on assessing the center of mass of total fluorescence. Consistent with studies of dispersed Golgi ministacks [31], we found that the Golgi polarization was blocked after BFA treatment.

Nevertheless, treatment with BFA did not influence GM1 polarization, although the expression and clustering pattern of GM1 were greatly altered. BFA treatment was characterized by overall decreased expression levels of GM1 and a propensity for clustering higher than expected from the low labeling density. Since Golgi-derived vesicles do not reach the plasma membrane under BFA treatment, the decreased levels of GM1 labeling suggests that the Golgi apparatus does play at least a partial role in supplying GM1 to the plasma membrane. This result suggests that the Golgi apparatus is responsible for synthesizing and delivering GM1 to the plasma membrane under normal conditions, while an alternative mechanism creates the polarized phenotype. The GM1 observed after BFA treatment was probably already present at the plasma membrane or in recycling pathways, and synthesis of new GM1 was halted by the drug treatment.

BFA may inhibit the delivery of other proteins or lipid species that are required for normal formation of membrane microdomains at the leading edge, including Apolipoprotein B, sphingomyelin and GD3, CD8 surface receptors, and influenza virus antigens [71]. Although sphingomyelin and GD3 are reportedly blocked by BFA, other studies discussed below classify these lipids as following BFA-insensitive routes to the cell surface, making interpretation unclear and contradictory. It is unknown why halting secretion from the Golgi apparatus via BFA treatment would lead to a degree of clustering higher than expected. An explanation might lie in a study using synthetic membranes composed of sphingomyelin, dioleoyl phosphatidylcholine and cholesterol to demonstrate that the addition of GM1 caused increased heterogeneity and decreased size of liquid ordered domains [79]. If GM1 acts to modulate lipid raft size, then BFA treatment leading to reduced levels of GM1 might induce larger clustered formations.

### Fast, Golgi-independent membrane polarization

We have shown that polarization of GM1 in the plasma membrane occurs before Golgi apparatus polarization and is not affected by either the position or functional state of the Golgi. Our results contradict in part the idea that material originating from the Golgi apparatus is responsible for establishing polarization of the plasma membrane. Previous experiments that suggested a primary role for the Golgi apparatus in asymmetric transport and membrane polarization relied on expression of VSVG, which does depend on the Golgi apparatus position to direct polarized membrane insertion [29], [37], [52]. Notably, Yadav et al. have described inhibiting Golgi polarization by dispersing the Golgi into functional ministacks, leaving secretion intact, which, inhibited the development of VSVG asymmetry in the plasma membrane [31]. An active sorting role for the Golgi apparatus has also been established for the asymmetric transport of lipid raft-associated GPI-anchored proteins and proteins modified with N- and O-glycans via microtubule motor protein interactions [80].

On the other hand, it has been proposed that the initial events of cell polarization originate at the plasma membrane, based on cell-cell and cell-substrate contacts, inducing cytoskeletal reorganization and, consequently, Golgi polarization, which functions in a stabilizing and reinforcing capacity [73]. Early plasma membrane polarization, and specifically the generation of plasma membrane potential via polarization of ion channels and pumps, has also been shown to directly modulate the actin cytoskeleton and drive other polarization events [81]. Other Golgi-independent mechanisms for achieving plasma membrane polarity have also been revealed, including self assembly and clustering [48], actin-mediated trapping [49], microtubule-based active transport [43], or asymmetric membrane insertion mediated by non-classical pathways [52], [82]–[84].

We observed fast, BFA-insensitive GM1 polarization that was dependent on PI3K, and activated by LPA exposure in leading edge cells. Many protein and lipid species associated with lipid rafts have been shown to follow non-classical, BFA-insensitive secretion, including cholesterol [52], [85], sphingomyelin [86]–[88], and the ganglioside GD3 [89]. Interestingly, all of these lipids follow similar kinetics to those demonstrated here for GM1, taking approximately 10 min to reach the plasma membrane. The intermediated compartment (IC), formed by a stable tubular structure at the interface between the ER and Golgi apparatus, is thought to play a central role in BFA-insensitive pathways by providing either a direct route to the plasma membrane or by bypassing the Golgi compartment and fusing with post-Golgi endocytic recycling compartments trafficked via microtubules to the plasma membrane. The precise mechanisms, specific coat proteins, and membrane fusion factors remain, however, unknown [82]. The observed BFA-insensitive polarization behavior of GM1 in the plasma membrane could suggest that the biosynthetic pathway of GM1 parallels that of cholesterol and sphingomyelin. However, a puzzling factor is that the total amount of GM1 in the plasma membrane was greatly reduced upon BFA treatment, implying at least partial reliance on classical, BFA-sensitive Golgi synthesis pathways.

Another explanation may lie in recent studies describing a novel system of endocytic membrane recycling pathways termed CLICs (clathrin-independent carriers) [83], [84]. CLICs are thought to represent a mechanism for bulk plasma membrane recycling that contributes to rapid changes in plasma membrane morphology. Intriguingly, CLICs are regulated by Cdc42, and wortmannin inhibits fusion of CLICs with early endosomes and thus the maturation of CLIC compartments. It might be the case that GM1 in the plasma membrane is sequestered into intracellular CLIC compartments until stimulation with polarization-inducing growth factors causes leading edge CLIC compartments to fuse with the plasma membrane. This scenario would explain our observation that wortmannin treated cells had significantly reduced GM1 content in the plasma membrane (Figure 5), while providing an attractive mechanism for achieving fast polarization of GM1.

### Role of the polarized Golgi apparatus

The question remains as to what function the polarized Golgi apparatus serves, if membrane asymmetry can be achieved independently. It could be that Golgi-derived vesicles carry specific proteins or lipid species essential for the regulation or modulation of plasma membrane polarity. The lack of these essential Golgi-derived proteins or lipid species might explain the aberrant clustering pattern of GM1 that we observed after BFA treatment inhibited Golgi exit. Cdc42 has a central role in the development and maintenance of cell polarization and migration, and active Cdc42 has been localized to the Golgi apparatus, binding to the γ subunit of the COP coatamer complex [90], [91]. Other Rho GTPases, including Rho B and H-Ras also localize to both the plasma membrane and the Golgi apparatus [92]. Cdc42 localization is BFA-sensitive and thought to regulate Golgi secretion pathways, but the exact role in this context is unknown [23], [93]. Activating Cdc42 through the expression of the oncogene Dbl causes a translocation of Cdc42 from the Golgi apparatus to newly formed lamellipodia in the plasma membrane [92]. Although to our knowledge it has never been directly shown, it may be possible that the Golgi apparatus is involved in directly trafficking Cdc42, and possibly other Rho proteins, to the plasma membrane during cell polarization and that the polarized localization assists in this function.

It is also possible that the polarized Golgi apparatus contributes to the later stages of polarization maintenance and cell migration. Blocking traffic from the Golgi apparatus has been known for some time to inhibit cell migration [33]–[35]. Our experiments demonstrate that inhibiting MEK/ERK with U0126 significantly inhibits two-dimensional cell migration after 48 h in a wound closure assay, although we cannot be sure whether these effects arise from blocking Golgi polarization or other downstream effects of MEK/ERK inhibition. That the Golgi apparatus plays its essential role in the later stages of cell migration rather than in the initial stages of establishing polarity is consistent with the idea that Golgi polarity acts in a stabilizing or reinforcing capacity [94], and, in any case, does not exclude the other possible roles described above.

Finally, the Golgi apparatus has also been shown to play an important role in cell signaling, and its polarized position within the cell could contribute to this role. Two kinases involved in cell polarization and migration, YSK1 and MST4, were shown to localize to the Golgi apparatus through interactions with Golgi matrix protein GM130, which directly activated the kinases [95]. Ras shows diverse activation kinetics and second messenger binding partners based on its localization to the plasma membrane or Golgi apparatus, and can even be activated independently in one location or the other [96]. Based on its role in cell signaling, it is possible that the polarized location of the Golgi apparatus contributes to the maintenance of intracellular gradients by localizing activated kinases like YSK1 and Ras towards the front of polarized cells.

### Biochemical and mechanistic separation in polarization response

We have demonstrated that the uncoupled polarization responses of the Golgi apparatus and GM1 in the plasma membrane depend on MEK and PI3K activation, respectively. The experiments indicate that the polarization events are controlled by separate biochemical pathways, yet migration is more efficient when both pathways are active. The MEK/ERK pathway seems to contribute more to the longer timescales of two-dimensional migration while the PI3K/AKT pathway contributes more to shorter timescales of three-dimensional matrix invasion.

Both the MEK/ERK and PI3K/AKT pathways are downstream of Cdc42, the master regulator of both cell migration and cell division [22], [23], [46]. With Cdc42 at the center of cell polarization events, it is consistent that activation of MEK/ERK and PI3K/AKT both follow from the activation of Cdc42, although it is remarkable that there seems to be little cross talk between these two downstream pathways, as evidenced by the fact that we can distinguish cellular responses based on the specific inhibitors used. This suggests that it may be advantageous for the cell to separately control the activation of individual pathways, and by extension, individual cell polarization events. We can speculate that the two divergent pathways controlling Golgi apparatus and GM1 polarization may function as a synergistic regulator of cell migration. Only when both pathways are activated and both systems engaged can migration proceed efficiently. The need for two separately engaged pathways may serve as a necessary regulation that constrains migration except for where strictly required.

Studies of the genetic destabilization found in cancer provide examples of inappropriate activation of the MEK/ERK and PI3K/AKT pathways. Both the Ras/Raf/MEK/ERK and the PI3K/AKT pathway are frequently mutated to constitutively active forms in many types of human cancers, and convey invasive properties [97], [98]. Consequently, the interplay between these separately regulated pathways has been a prime target for cancer therapeutics. Cancerous cells have been shown to take advantage of the uncoupled nature of the pathways; where one pathway is inhibited by chemotherapeutic drugs, the other seems to compensate to drive cell proliferation and migration [99], [100]. A clinical trial combining inhibition of MEK and AKT showed more effective tumor reduction and increased positive outcomes than inhibition of a single pathway alone [101]. Further research into the mechanisms involved in cell polarization and migration, and the interplay between cellular pathways and systems will contribute to the development of alternative strategies to effectively treat an array of diseases, including metastases in cancer.

## Materials and Methods

### Cell culture and scratch assay for cell polarization

ECV304 human epithelial [102] cultured cells were purchased from ATCC and provided to us by Dr. Paola Defilippi [103]. The LNCaP human prostate cancer cell line was purchased from ATCC and provided to us by Dr. Chiara Lanzuolo. ECV304 cells were grown in DME/F12 1∶1 media (Invitrogen) and LNCaP cells were grown in RPMI-1640 media (Invitrogen). The media from both cell lines was supplemented with penicillin (100 U/ml), streptomycin (100 µg/ml), and 10% FBS (all Invitrogen) in a humidified chamber at 5% CO_2_ and 37°C. For scratch experiments, ECV304 cells were plated on 18 mm No.1 thickness glass cover slips (VWR) and allowed to grow to confluence, after which cells were starved overnight with antibiotic- and serum-free DME/F12. A wound edge was created with a sterilized razor blade by removing the cells from one half of the cover slip. The sample was allowed to recover for 2 h, after which cells were stimulated with 2 µM LPA (Sigma Aldrich) at 37°C for the times indicated. Drugs were added as indicated with the following concentrations: 10 µM U0126, 100 nM wortmannin (Invitrogen), and 5 µg/ml BFA (Sigma Aldrich). All drug treatments consisted of a 30 min pre-incubation in the cell culture incubator at 37°C, after which LPA was added in the continued presence of the drug. Stimulation and drug treatments were performed on unlabeled live ECV304 cells to avoid aggregation effects of cholera toxin subunit B, and to avoid any possibility of affecting plasma membrane diffusion rates of labeled molecules.

### Fixation and labeling

We implemented a protocol of two separate rounds of fixation and staining that was used for all experiments to prevent non-specific binding of QDots. First, cover slips were fixed with 1.5% PFA (Sigma Aldrich) for 10 min at room temperature, washed with PBS, blocked with 1 mg/ml BSA (Sigma Aldrich), and incubated for 10 min with 1 µg/ml cholera toxin subunit B conjugated to biotin (Sigma Aldrich C9972), followed by washing and incubation for 1 min with QD 655 functionalized with streptavidin (Invitrogen) diluted 1∶1000 in QD binding buffer [104]. We then permeabilized the cells by submerging cover slips in −20°C methanol (ACS, ISO ≥99.8%, Sigma Aldrich) for 15 min. After rehydration in PBS and blocking with BSA, we stained the Golgi apparatus with a mouse monoclonal primary antibody directed towards GM130 (TLGM130; 1∶200; Transduction Labs No. 610822, BD Biosciences), followed by a secondary goat anti-mouse antibody conjugated to Alexafluor 488 (1∶300; Invitrogen). As a final step, we incubated cover slips for 1 min with 1 µg/ml Hoechst 33342 (Invitrogen). Washing was performed between each step in PBS. The cover slips were mounted on glass microscope slides with a Mowiol 4–88 solution (9% Mowiol w/w, 22.7% glycerol w/w, 0.2 M Tris HCl pH 8.5, 3 mM NaN_3_, VWR), taking care to orient the wound edge parallel to the length of the slide.

### Image collection and processing

A Nikon Eclipse TE300 inverted microscope was utilized with a Nikon Plan-Apo 60XA/1.40 n.a. oil immersion objective. Excitation light was provided by a mercury lamp (Hg100W). Fluorescence signal was detected by a Hamamatsu Photonics K.K. Japan high sensitivity silicon-intensified target (SIT) camera, coupled to a Hamamatsu Argus Image Processor and a Hamamatsu C2400 camera controller. The Argus Image Processor was set to average 8 frames of the standard 30 frames per second camera output in order to improve signal-to-noise and reduce the visible blinking effects of QDots. Digital images were captured using a custom-made Virtual Instrument produced with Labview 7.1.

Images were collected along the scratch surface. Prior to capturing digital images of the wound edge cells, we carefully aligned the coverslip so that the wound was parallel to the *x*-axis of both the microscope stage and the camera's ccd by scanning back and forth and adjusting the slide manually. In this way, our images always had the wound in the upper quadrant, and the positive *y*-axis facing the wound was used as a reference to calculate polarization. Three QD images representing different focal layers of the plasma membrane were transformed into a maximum intensity projection that was combined with the Golgi apparatus and Hoechst images into an RGB composite. Regions of interests (ROIs) were drawn around individual cells based on the RGB composites. Image processing was performed using the program ImageJ64 v1.43u. Background fluorescence was subtracted using the Brightness and Contrast function in ImageJ by increasing the minimum threshold until the background read as 0. Using center of mass measurements requires that the background of an image or ROI be reduced to 0 because even low levels of background noise do not allow an accurate calculation of the center of mass. Care was taken not to over aggressively reduce the background by assuring that signals from single QDs remained in the final image. Representative images were cropped and levels adjusted uniformly in Photoshop CS4 during the compilation of figures.

### Quantification of GM1 and Golgi apparatus polarization

GM1 polarization was determined by comparing the weighted center of mass from QD fluorescence distribution in the plasma membrane with the center of mass calculated for the area of the cell. Using the previously established ROI, the center of mass of the plasma membrane QD fluorescence from a single cell (C_m PM_) was measured in ImageJ. All pixels within the ROI were then set to 1, and the geometric center of mass of the cell (C_m cell_) was measured. We defined plasma membrane polarization according to the variable ΔY, calculated from subtracting the *y* coordinate of the C_m PM_ from the *y* coordinate of the C_m cell_.
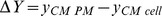
where *y*_CM PM_ and *y*_CM cell_ are the *y* coordinates of C_m PM_ and C_m cell_, respectively.

To calculate the Golgi angle, the centers of mass of the nucleus and Golgi apparatus fluorescence were measured sequentially in ImageJ and used to determine coordinates of the nucleus-to-Golgi vector as follows:
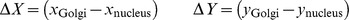
where ΔX and ΔY are coordinates of the nucleus-to-Golgi vector with origin at (0,0), *x*_Golgi_ and *y*_Golgi_ are the *x* and *y* coordinates of the Golgi center of mass and *x*_nucleus_ and *y*_nucleus_ are the *x* and *y* coordinates of the nucleus center of mass. The Golgi angle was calculated from the vector coordinates as follows:



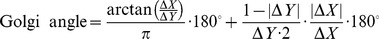
where G_angle_ is the Golgi angle, and ΔX and ΔY are the coordinates of the vector defined above. The second factor in the equation enables measurement of the defined angle on all four quadrants of the coordinate system we adapted, breaking the symmetry of the arctan function. Background noise was subtracted as described above prior to center of mass measurements.

### GM1 clustering analysis

Flattened z stack images of QDot labeled GM1 were subjected to particle analysis in ImageJ. Background was subtracted using a rolling radius of 25 pixels, and images were thresholded below a value of 29, which was determined empirically to retain a signal for single QDots in the majority of images. Particle analysis was run on previously determined single cell ROIs with no constraints on particle size or circularity, and the number, average size, area fraction, and total area of the cell were returned as raw data. Labeling density is equivalent to the area fraction, or the percent of the total cell area covered by fluorescent signal after thresholding. The average size of aggregates in µm^2^ was converted from the output in ImageJ particle analysis in square pixels.

### Cell migration and invasion assays

For two-dimensional cell migration assays, ECV304 cells were grown as described above were plated in 6 well plates on glass cover slips. When the cells reached confluence, they were starved overnight in antibiotic- and serum-free DME/F12 before a scratch wound was inflicted using a 20–200 µl pipette tip. Images were collected of the 0 h time point using a 10 X objective and inverted microscope. Where indicated, drugs were added for a preincubation period of 30 min at 37°C. LPA was then added at a concentration of 2 µM, and cells were placed in a 5% CO_2_ incubator at 37°C. After 24 h and again after 48 h, cells were removed from the incubator and imaged using the same 10 X objective and inverted microscope. Care was taken to align the scratch along the *y* axis of the camera to aid subsequent image quantification.

For three-dimensional migration, both ECV304 and LNCaP cells were grown as described above. Matrigel invasion assays were performed in 24 well plates using 0.8 µm pore PET inserts (BD Biosciences). On the day of the experiment, matrigel basement membrane matrix, growth factor reduced (BD Biosciences) was diluted to a final concentration of 300 µg/ml in serum-free media and 100 µl was applied to the pore surface. After an incubation at 37°C for 30 min, the matrigel-loaded inserts were placed in 800 µl of media in the lower chamber of a 24 well plate, containing 2 µM LPA and inhibitory drugs where indicated. Finally, cells were dispersed with TE solution (0.25% trypsin and 0.53 mM EDTA in PBS), counted with a hemocytometer, and brought to a final concentration of 1×10^5^ cells/ml. 300 µl of the cell solution was added to the top chamber of each matrigel insert for a final count of 30,000 cells/well. Immediately after adding the cells, inhibitory drugs were added to the top chamber as well. Cells were incubated for 20 h, then fixed in 4% para-formaldehyde solution for 2 min followed by permeabilization with room temperature methanol for 20 min and staining with Trypan blue diluted 1∶5 in PBS, washing with PBS between every step. Finally, a cotton swab was used to remove cells from the surface of the 0.8 µm pore membrane corresponding to the top chamber, leaving intact only those cells which had migrated to the bottom surface. After a brief drying period, the whole insert was placed on a cover slide and imaged with a 10x objective on an inverted microscope.

### Statistical analysis

Experiments were repeated a minimum of three times. Values of ΔY and the Golgi angle from at least three independent experiments were pooled. N values reported in the figure legends represent the total number of cells analyzed over three or more experiments. We analyzed cumulative distributions of both membrane polarization data (ΔY) and Golgi angle data with Kolmogorov-Smirnov non-parametric tests, and p values of <0.05 were considered significant. Correlation analysis was performed for coupled values of ΔY and the Golgi angle collected from single cells. Spearman correlation tests were used to assess significance, and the Spearman's rank correlation coefficient ρ and the associated p value are reported, with p<0.05 regarded as significant. For the wound healing assay, the area of the scratch was measured at various points along the scratch during at least three independent experiments. The results were pooled for each experimental condition, and two-way ANOVA and Bonferroni post-tests comparing all columns were applied to assess significance. Similarly, for matrigel invasion assays, migrated cells were counted for several frames per experiment, and the results for at least three independent experiments were pooled and subjected to analysis by one way ANOVA with Tukey post-tests to compare all experimental conditions. N values represent the total number of measurements, either scratch area or number of cells per frame.

## Supporting Information

Figure S1 **Comparison of classical and semi-automated methods for measuring Golgi apparatus polarization.** The percentages of cells measured with the semi-automated method whose Golgi angle fell between −60° and +60° are shown as patterned bars. The percentages of cells scored by the classical method as having polarized Golgi (a Golgi that falls within a 120° angle facing the wound edge) is shown as solid bars. (**A**) 10 min stimulation with either serum, EGF, or LPA, does not lead to significant Golgi polarization compared to control “none,” and no significant differences were found between the two methods of assessing Golgi polarization. (**B**) 30 min stimulation with either serum, EGF, or LPA, leads to significant Golgi polarization levels compared to control, and no significant differences were present between the two evaluation methods. The means of the results of three individual experiments are presented with error bars representing the standard error of the mean (SEM), and Student's t tests were performed to assess significance, with p≤0.05 considered significant.
(TIF)

Figure S2 **Stimulations with FBS, EGF, or LPA yield similar results.** In addition to LPA, we looked at the effects of FBS (1%) and EGF (2 ng/ml) for all conditions tested: 10 min stimulation (**A–B**), 30 min stimulation (**C–D**), pretreatment and concurrent stimulation with U0126 (**E–F**), BFA (**G–H**), and wortmannin (**I–J**). ΔY in µm and the absolute value of the Golgi angle are plotted as cumulative distributions and analyzed by Kolmogorov-Smirnov statistical tests. Drug-treated conditions were compared with the baseline control “none” and with the stimulated, drug-free control (denoted by brackets where applicable). *** represents p≤0.001, ** represents p≤0.01, and * represents p≤0.05.
(TIF)

File S1 **Tables S1, S2, and S3 include two-way ANOVA Boneferroni post-test results for the time points 0 h, 24 h, and 48 h of the wound healing assay. Tables S3 and S4 represent the one-way ANOVA Tukey post-test results for ECV304 Matrigel invasion assay.**
(DOCX)
