# Supplementary figures and images for: Decoupling Polarization of the Golgi Apparatus and GM1 in the Plasma Membrane
Source: PLoS One. 2013 Dec 2;8(12):e80446. doi: 10.1371/journal.pone.0080446 (PMC3846482; doi:10.1371/journal.pone.0080446)

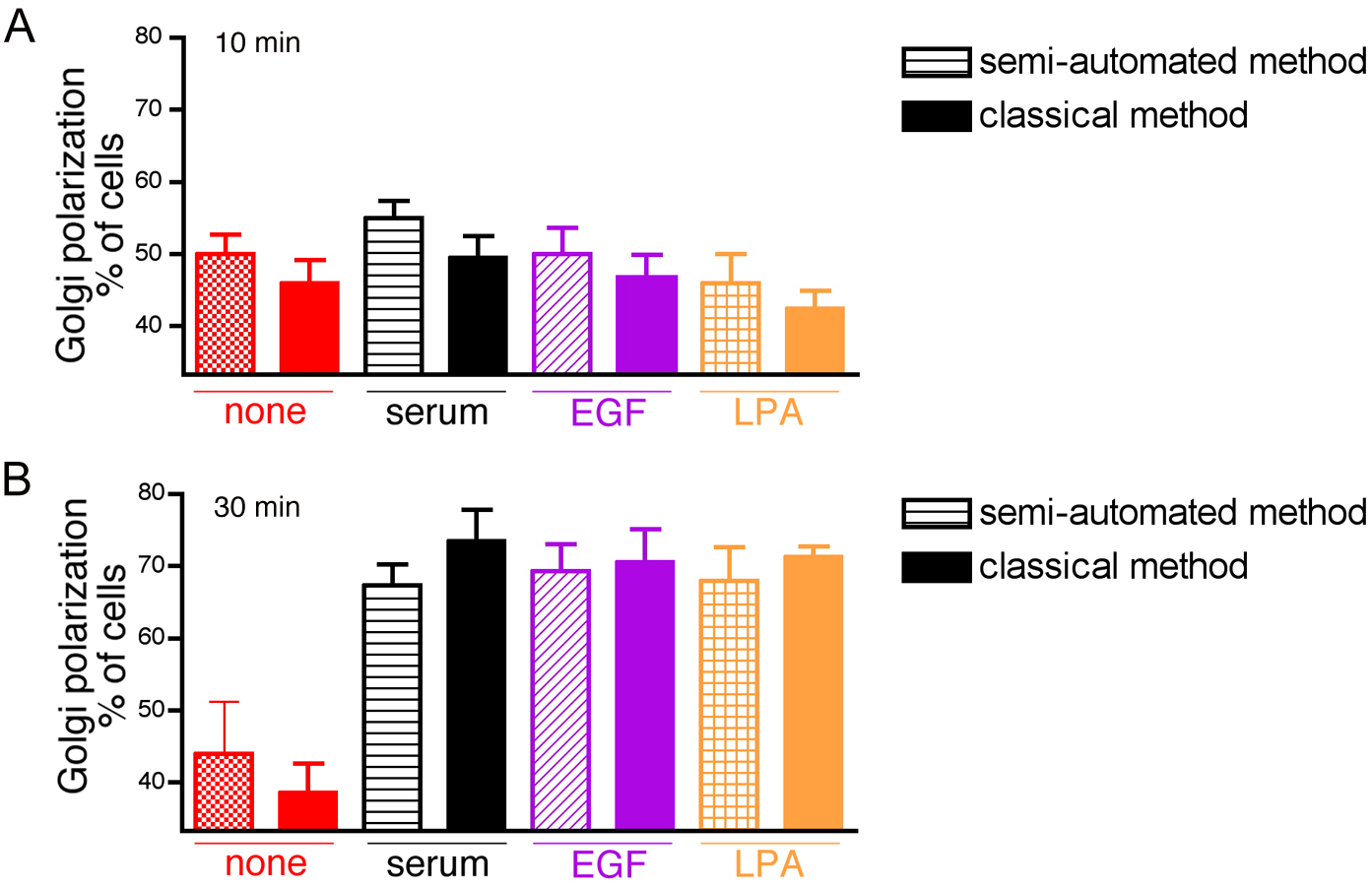

Supplement: Figure S1 — Comparison of classical and semi-automated methods for measuring Golgi apparatus polarization. The percentages of cells measured with the semi-automated method whose Golgi angle fell between −60° and +60° are shown as patterned bars. The percentages of cells scored by the classical method as having polarized Golgi (a Golgi that falls within a 120° angle facing the wound edge) is shown as solid bars. (A) 10 min stimulation with either serum, EGF, or LPA, does not lead to significant Golgi polarization compared to control “none,” and no significant differences were found between the two methods of assessing Golgi polarization. (B) 30 min stimulation with either serum, EGF, or LPA, leads to significant Golgi polarization levels compared to control, and no significant differences were present between the two evaluation methods. The means of the results of three individual experiments are presented with error bars representing the standard error of the mean (SEM), and Student's t tests were performed to assess significance, with p≤0.05 considered significant. (TIF) [file pone.0080446.s001.tif]

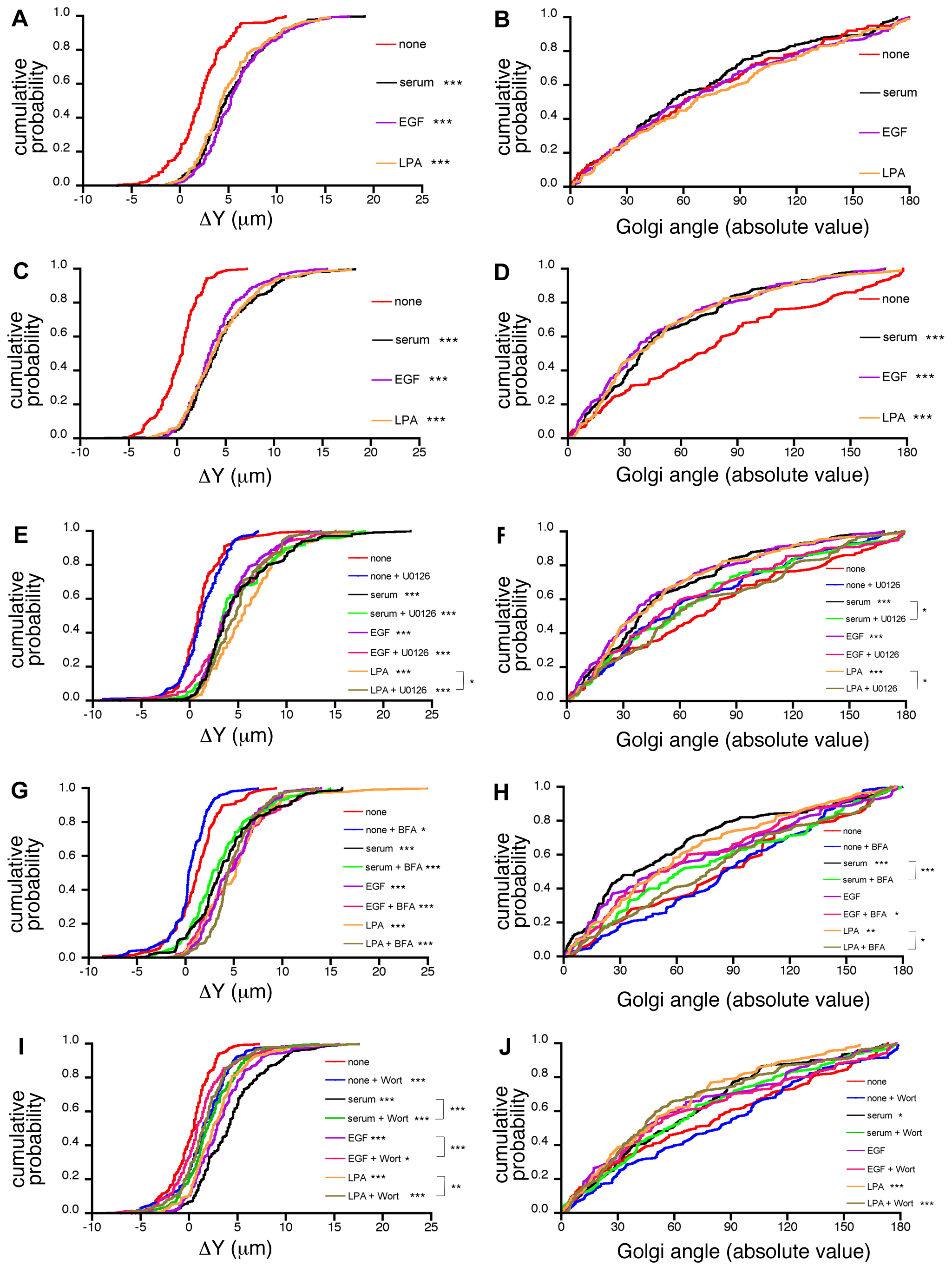

Supplement: Figure S2 — Stimulations with FBS, EGF, or LPA yield similar results. In addition to LPA, we looked at the effects of FBS (1%) and EGF (2 ng/ml) for all conditions tested: 10 min stimulation (A–B), 30 min stimulation (C–D), pretreatment and concurrent stimulation with U0126 (E–F), BFA (G–H), and wortmannin (I–J). ΔY in µm and the absolute value of the Golgi angle are plotted as cumulative distributions and analyzed by Kolmogorov-Smirnov statistical tests. Drug-treated conditions were compared with the baseline control “none” and with the stimulated, drug-free control (denoted by brackets where applicable). *** represents p≤0.001, ** represents p≤0.01, and * represents p≤0.05. (TIF) [file pone.0080446.s002.tif]
