# Supplementary material for: Decoupling Polarization of the Golgi Apparatus and GM1 in the Plasma Membrane
Source: PLoS One. 2013 Dec 2;8(12):e80446. doi: 10.1371/journal.pone.0080446 (PMC3846482; doi:10.1371/journal.pone.0080446)
Supplement: File S1 — Tables S1, S2, and S3 include two-way ANOVA Boneferroni post-test results for the time points 0 h, 24 h, and 48 h of the wound healing assay. Tables S3 and S4 represent the one-way ANOVA Tukey post-test results for ECV304 Matrigel invasion assay. (DOCX) [file pone.0080446.s003.docx]

Table S1 Two way ANOVA Boneferroni post-test results for 0h time point of wound healing assay

| 0h | None | LPA | U0126 | Wort | Wort + U0126 | BFA |
| --- | --- | --- | --- | --- | --- | --- |
| None N=9 | - | p>0.05 (n.s.) | p>0.05 (n.s.) | p>0.05 (n.s.) | p>0.05 (n.s.) | p>0.05 (n.s.) |
| LPA N=12 |  | - | p>0.05 (n.s.) | p<0.01 (**) | p>0.05 (n.s.) | p>0.05 (n.s.) |
| U0126 N=11 |  |  | - | p>0.05 (n.s.) | p>0.05 (n.s.) | p>0.05 (n.s.) |
| Wort N=10 |  |  |  | - | p<0.01 (**) | p<0.05 (*) |
| Wort + U0126 N=11 |  |  |  |  | - | p>0.05 (n.s.) |
| BFA N=10 |  |  |  |  |  | - |

Table S2 Two way ANOVA Boneferroni post-test results for 24h time point of wound healing assay

| 24h | None | LPA | U0126 | Wort | Wort + U0126 | BFA |
| --- | --- | --- | --- | --- | --- | --- |
| None N=13 | - | p<0.001 (***) | p<0.01 (**) | p<0.001 (***) | p<0.001 (***) | p<0.001 (***) |
| LPA N=17 |  | - | p<0.001 (***) | p<0.001 (***) | p<0.001 (***) | p<0.001 (***) |
| U0126 N=13 |  |  | - | p>0.05 (n.s.) | p>0.05 (n.s.) | p<0.001 (***) |
| Wort N=15 |  |  |  | - | p>0.05 (n.s.) | p<0.001 (***) |
| Wort + U0126 N=15 |  |  |  |  | - | p<0.001 (***) |
| BFA N=12 |  |  |  |  |  | - |

Table S3 Two way ANOVA Boneferroni post-test results for 48h time point of wound healing assay

| 48h | None | LPA | U0126 | Wort | Wort + U0126 | BFA |
| --- | --- | --- | --- | --- | --- | --- |
| None N=12 | - | p<0.001 (***) | p<0.01 (**) | p<0.001 (***) | p<0.001 (***) | p<0.001 (***) |
| LPA N=14 |  | - | p<0.001 (***) | p>0.05 (n.s.) | p<0.001 (***) | p<0.001 (***) |
| U0126 N=13 |  |  | - | p<0.001 (***) | p>0.05 (n.s.) | p<0.001 (***) |
| Wort N=13 |  |  |  | - | p<0.01 (**) | p<0.001 (***) |
| Wort + U0126 N=11 |  |  |  |  | - | p<0.001 (***) |
| BFA N=10 |  |  |  |  |  | - |

Table S4 One way ANOVA Tukey post-test results for ECV304 Matrigel invasion assay

| ECV304 cells | None | LPA | U0126 | Wort | Wort + U0126 | BFA |
| --- | --- | --- | --- | --- | --- | --- |
| None N=56 | - | p<0.001 (***) | p<0.001 (***) | p<0.001 (***) | p<0.001 (***) | p>0.05 (n.s.) |
| LPA N=53 |  | - | p>0.05 (n.s.) | p<0.001 (***) | p<0.001 (***) | p<0.001 (***) |
| U0126 N=59 |  |  | - | p<0.001 (***) | p<0.001 (***) | p<0.001 (***) |
| Wort N=64 |  |  |  | - | p>0.05 (n.s.) | p<0.05 (*) |
| Wort + U0126 N=59 |  |  |  |  | - | p<0.01 (**) |
| BFA N=54 |  |  |  |  |  | - |

Table S5 One way ANOVA Tukey post-test results for LNCaP Matrigel invasion assay

| LNCaP cells | None | LPA | U0126 | Wort | Wort + U0126 | BFA |
| --- | --- | --- | --- | --- | --- | --- |
| None N=32 | - | p<0.001 (***) | p>0.05 (n.s.) | p<0.05 (*) | p<0.05 (*) | p<0.05 (*) |
| LPA N=24 |  | - | p<0.001 (***) | p<0.001 (***) | p<0.001 (***) | p<0.001 (***) |
| U0126 N=42 |  |  | - | p>0.05 (n.s.) | p>0.05 (n.s.) | p>0.05 (n.s.) |
| Wort N=39 |  |  |  | - | p>0.05 (n.s.) | p>0.05 (n.s.) |
| Wort + U0126 N=52 |  |  |  |  | - | p>0.05 (n.s.) |
| BFA N=38 |  |  |  |  |  | - |
